# Supplementary material for: Switching of the Chiral Magnetic Domains in the Hybrid Molecular/Inorganic Multiferroic (ND4)2[FeCl5(D2O)]
Source: Sci Rep. 2018 Jul 13;8:10665. doi: 10.1038/s41598-018-28883-z (PMC6045669; doi:10.1038/s41598-018-28883-z)
Supplement: Supplementary file 1 — Supporting Information [file 41598_2018_28883_MOESM1_ESM.pdf]

Supporting Information:

Switching of the Chiral Magnetic Domains in the Hybrid Molecular/Inorganic Multiferroic $(ND_4)_2[FeCl_5(D_2O)]$

J. Alberto Rodríguez-Velamazán,[1*] Oscar Fabelo,[1*] Javier Campo,[2] Juan Rodríguez-Carvajal,[1] Navid Qureshi[1] and Laurent C. Chapon[1,3]

[1] Institut Laue-Langevin, 71 Avenue des Martyrs, CS 20156, 38042 Grenoble Cedex 9, France.

[2] Instituto de Ciencia de Materiales de Aragón, CSIC-Universidad de Zaragoza, C/ Pedro Cerbuna 12, E-50009, Zaragoza, Spain.

[3] Diamond Light Source Ltd, Harwell Sci & Innovat Campus, Didcot OX11 0DE, Oxon, England

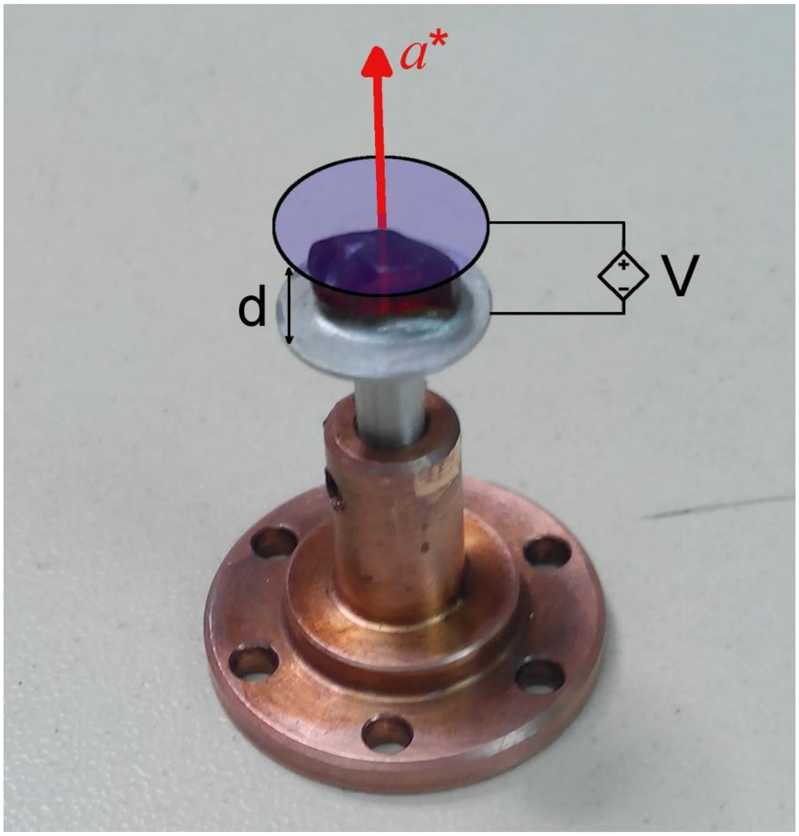

FIG. S1. Single crystal of $(ND_4)_2[FeCl_5(D_2O)]$ used in the spherical neutron polarimetry experiments, mounted in the sample support designed for the application of electric field by a potential difference between two parallel horizontal aluminum plates. The crystal was fixed to the lower plate by silver epoxy with the *a**-axis in the vertical direction, and the upper plate positioned at ca. 1 mm from the sample surface (total distance between electrodes, d = 3.15 mm, sample thickness, ca. 2 mm).

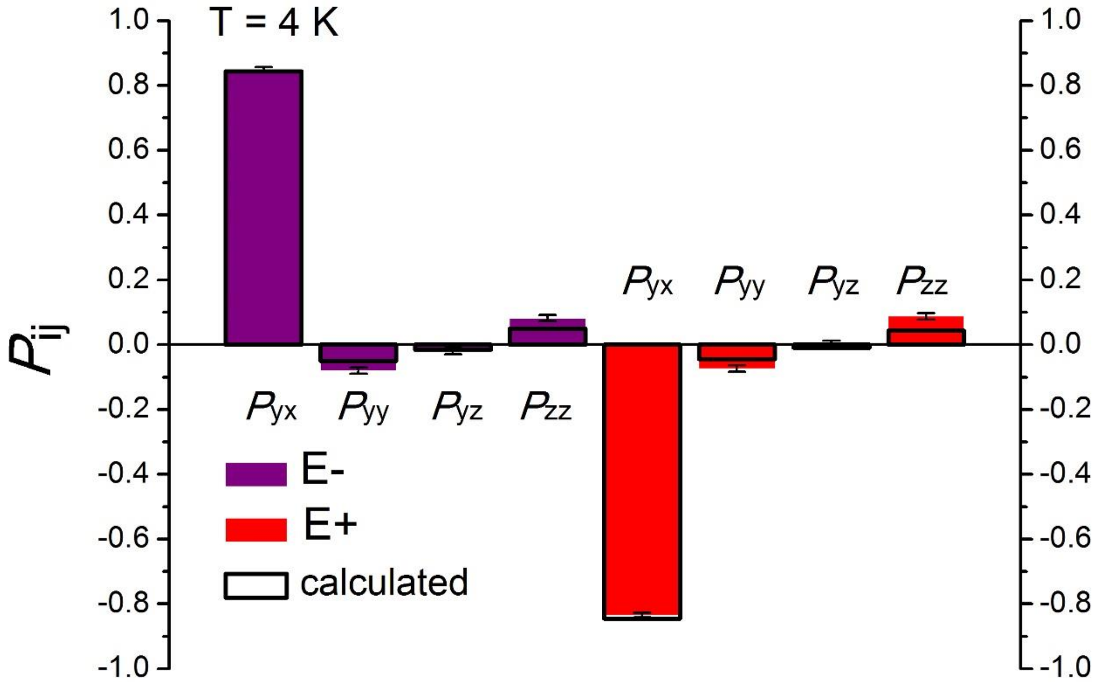

FIG. S2. Observed (solid color columns and error bars) and calculated (black rectangles) neutron spherical polarization matrix elements, P_{ij}, for the (0 1 -0.23) magnetic reflection of a $(ND_4)_2[FeCl_5(D_2O)]$ crystal oriented with *a** parallel to the z-axis. Results corresponding to the cycloidal phase (T = 4 K) for the sample cooled under negative (purple) and positive (red) electric field of 25 kV cm^{-1} applied along *a**. The calculated values are the results of the joint fit of neutron spherical polarimetry and unpolarized neutron diffraction intensities.

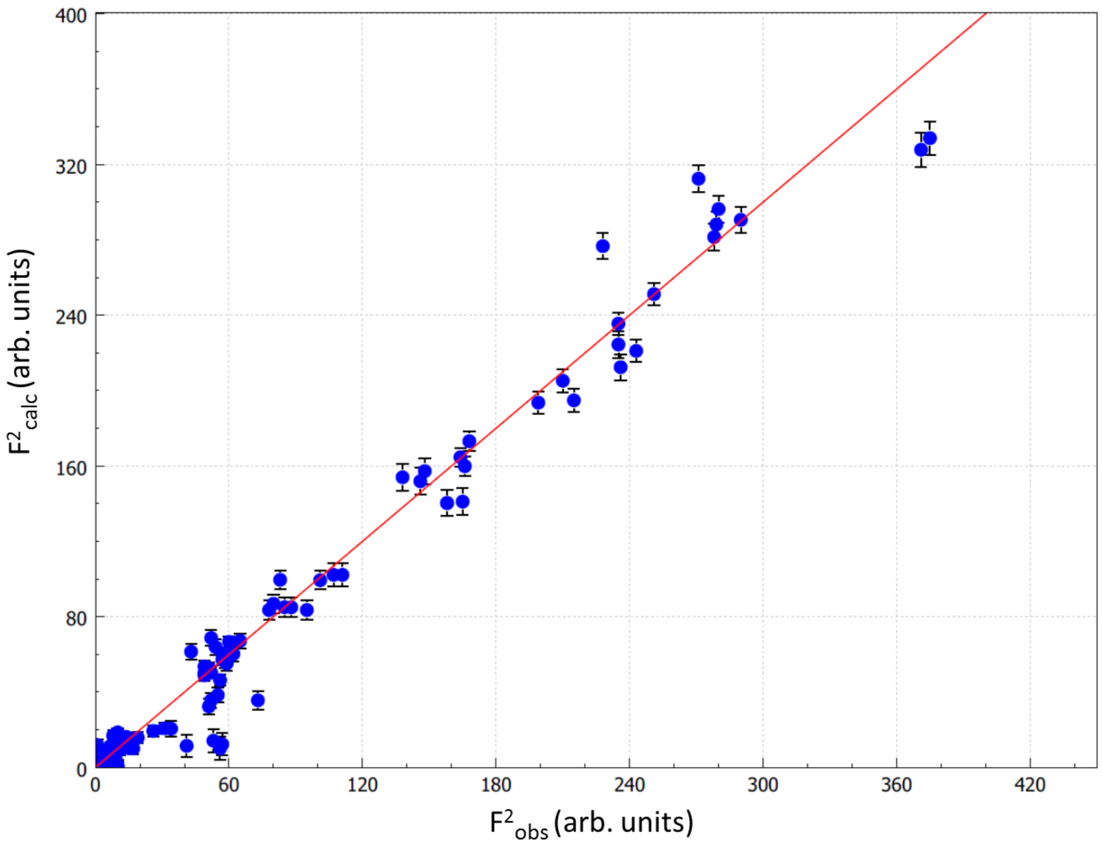

FIG. S3. Results of the joint fit of neutron spherical polarimetry and unpolarized neutron diffraction intensities: Plot of the observed *vs* calculated squared magnetic interaction vectors (here $F^2=|M_\perp|^2$) for unpolarized neutron diffraction (the experimental data are represented as blue circles and the ideal case $F^2_{cal} = F^2_{obs}$, is represented as a solid red line).

Summary of equations governing the spherical neutron polarimetry technique

The Blume-Maleyev equations describe the scattering of polarised neutrons. We adopt here a simplified crystallographic notation in order to make clear the important terms. We suppose that we are in the more general case of a crystal with a magnetic structure of arbitrary complexity that has some magneto-elastic coupling and there is a nuclear contribution to the magnetic reflections. We write the Blume-Maleyev equations for a reflection **h**=**H**+**k**, where **H** is a vector of the average crystallographic reciprocal lattice and the propagation vector **k** index the magnetic satellite. We shall call $I_{\mathbf{h}}$ the intensity (scattering cross-section) of the diffracted beam and, if the incident and scattered polarisations are noted as $\mathbf{P}_i$ and $\mathbf{P}_f$, respectively, the equations are:

Cross-section (Equation 1):

$$I_{\mathbf{h}} = N_{\mathbf{h}}N_{\mathbf{h}}^{*} + \mathbf{M}_{\perp\mathbf{h}}\mathbf{M}_{\perp\mathbf{h}}^{*} + \left(N_{\mathbf{h}}\mathbf{M}_{\perp\mathbf{h}}^{*} + N_{\mathbf{h}}^{*}\mathbf{M}_{\perp\mathbf{h}}\right)\mathbf{P}_i + i\left(\mathbf{M}_{\perp\mathbf{h}}^{*} \times \mathbf{M}_{\perp\mathbf{h}}\right)\mathbf{P}_i$$

Final polarisation (Equation 2):

$$\begin{aligned}\mathbf{P}_f I_{\mathbf{h}} = {} & N_{\mathbf{h}}N_{\mathbf{h}}^{*}\mathbf{P}_i - \left(\mathbf{M}_{\perp\mathbf{h}}\mathbf{M}_{\perp\mathbf{h}}^{*}\right)\mathbf{P}_i + \left(\mathbf{P}_i\ \mathbf{M}_{\perp\mathbf{h}}^{*}\right)\mathbf{M}_{\perp\mathbf{h}} + \left(\mathbf{P}_i\ \mathbf{M}_{\perp\mathbf{h}}\right)\mathbf{M}_{\perp\mathbf{h}}^{*} - \\ & - i\left(N_{\mathbf{h}}\mathbf{M}_{\perp\mathbf{h}}^{*} - N_{\mathbf{h}}^{*}\mathbf{M}_{\perp\mathbf{h}}\right)\times\mathbf{P}_i + \\ & + N_{\mathbf{h}}\mathbf{M}_{\perp\mathbf{h}}^{*} + N_{\mathbf{h}}^{*}\mathbf{M}_{\perp\mathbf{h}} - i\left(\mathbf{M}_{\perp\mathbf{h}}^{*} \times \mathbf{M}_{\perp\mathbf{h}}\right)\end{aligned}$$

Where the nuclear structure factor of reflection **h** is $N_{\mathbf{h}}$ and $\mathbf{M}_{\perp\mathbf{h}}$ is the magnetic interaction vector: perpendicular component of the magnetic structure factor to the scattering vector.

The four terms in the scattering cross section are called, respectively: nuclear contribution, magnetic contribution, nuclear-magnetic interference term and chiral term. For a non-polarised beam $\mathbf{P}_i$ =0 only the first two terms contribute to the diffraction pattern. For a pure magnetic reflection only the second and fourth terms are different from zero.

These equations are written in absolute form. They are independent from the particular frame to describe the vectorial quantities. Notice that in the expression of the scattered polarisation the last line regroups the terms that are independent from the incident polarisation. These are the terms that are exploited to obtain polarised beams from an initial non-polarised beam.
We shall consider in the following that we use an arbitrary Cartesian system for referring the component of the vectorial quantities. Let us concentrate in the expression of the scattered polarisation that we will write in a tensorial form. We shall drop the **h** and ⊥ indices to simplify the notation so that the expression of the final polarisation is:

$$\begin{aligned}\mathbf{P}_f I = {} & NN^{*}\mathbf{P}_i - \left(\mathbf{M}\,\mathbf{M}^{*}\right)\mathbf{P}_i + \left(\mathbf{P}_i\ \mathbf{M}^{*}\right)\mathbf{M} + \left(\mathbf{P}_i\ \mathbf{M}\right)\mathbf{M}^{*} - i\left(N\mathbf{M}^{*} - N^{*}\mathbf{M}\right)\times\mathbf{P}_i + \\ & + N\mathbf{M}^{*} + N^{*}\mathbf{M} - i\left(\mathbf{M}^{*}\times\mathbf{M}\right)\end{aligned}$$

The first line can be written in a matrix form and the second is just an added vector. Dividing by the cross-section we can write:

$$\mathbf{P}_f = \overleftrightarrow{P}\,\mathbf{P}_i + \mathbf{P}_c$$

with $$\mathbf{P}_c = \frac{N\mathbf{M}^* + N^*\mathbf{M} - i\left(\mathbf{M}^* \times \mathbf{M}\right)}{I} = \frac{\mathbf{W}_R - \mathbf{T}}{I}$$

being this term independent of the initial polarisation (except for the dependence of I) and it is usually called *created* polarization. Let us call the complex vector $\mathbf{W}= 2\,N\mathbf{M}^*= \mathbf{W}_R+i\,\mathbf{W}_I$ the *nuclear-magnetic interference vector*. We can see that $2\mathbf{W}_R=\mathbf{W}+\mathbf{W}^*=2(N\mathbf{M}^*+ N^*\mathbf{M})$ is a real vector and $2\mathbf{W}_I i=\mathbf{W}-\mathbf{W}^*= 2(N\mathbf{M}^*- N^*\mathbf{M})$ is a pure imaginary vector. The vector $\mathbf{M}^*\times \mathbf{M}$ is purely imaginary, so that $\mathbf{T} = i(\mathbf{M}^*\times \mathbf{M})$ is a real vector that we shall call hereafter the *chiral vector*.
In the Blume Cartesian reference system x is along the scattering vector, z is perpendicular to the scattering plane pointing up in the instrument and y completes the right handed system. In this system the intensity reduces to:

$$I = NN^* + \mathbf{MM}^* + \mathbf{W}_R\mathbf{P}_i + \mathbf{T}\,\mathbf{P}_i = I_N + I_M + \left(\mathbf{W}_R + \mathbf{T}\right)\mathbf{P}_i$$

$$I = I_N + I_M + T_x P_{ix} + W_{Ry} P_{iy} + W_{Rz} P_{iz}$$

And the matrix equation for polarization is:

$$\mathbf{P}_f = \frac{1}{I}\begin{pmatrix} I_N - I_M & -W_{Iz} & W_{Iy} \\ W_{Iz} & I_N + I_M^y - I_M^z & M_{mix} \\ -W_{Iy} & M_{mix} & I_N - I_M^y + I_M^z \end{pmatrix}\begin{pmatrix} P_{ix} \\ P_{iy} \\ P_{iz} \end{pmatrix} + \frac{1}{I}\begin{pmatrix} -T_x \\ W_{Ry} \\ W_{Rz} \end{pmatrix}$$

where we have put $I_M = \mathbf{M}.\mathbf{M}^* = M_x M_x^* + M_y M_y^* + M_z M_z^* = I_M^x + I_M^y + I_M^z = I_M^y + I_M^z$; the term along x disappears by definition of the reference frame. And we have called $M_{mix} = M_y^* M_z + M_y M_z^* = 2\,\mathrm{Re}(M_y M_z^*)$. The chiral vector is

$$\mathbf{T} = i(\mathbf{M}^* \times \mathbf{M}) = (T_x,\, 0,\, 0) = i(M_y M_z^* - M_y^* M_z,\, 0,\, 0) = -2(\mathrm{Im}(M_y M_z^*),\, 0,\, 0)$$

In practice the incident polarisation is put along x, $\boldsymbol{P}_i$=(1,0,0), y, $\boldsymbol{P}_i$=(0,1,0) and z, $\boldsymbol{P}_i$=(0,1,0), then we measure the three components of the scattered polarisation for each case, so that 9 numbers are obtained for a single reflection. Let us call I^α the scattered intensity when the incident polarisation is along α ($\alpha = x, y, z$). The nine numbers written in the form of a matrix are

$$P = \begin{pmatrix} \frac{I_N - I_M - T_x}{I^x} & \frac{W_{Iz} + W_{Ry}}{I^x} & \frac{W_{Rz} - W_{Iy}}{I^x} \\ \frac{-W_{Iz} - T_x}{I^y} & \frac{I_N + I_M^y - I_M^z + W_{Ry}}{I^y} & \frac{M_{mix} + W_{Rz}}{I^y} \\ \frac{W_{Iy} - T_x}{I^z} & \frac{M_{mix} + W_{Ry}}{I^z} & \frac{I_N - I_M^y + I_M^z + W_{Rz}}{I^z} \end{pmatrix}$$

In which each row corresponds to the final polarization when the initial polarization is along x, y and z. For the case of a pure magnetic reflection $\mathbf{W}$=0, I_N=0, $I^x = I_M + T_x$, $I^y = I^z = I_M$, so the matrix reduces to:

$$P_{mag} = \begin{pmatrix} -1 & 0 & 0 \\ \frac{-T_x}{I_M} & \frac{I_M^y - I_M^z}{I_M} & \frac{M_{mix}}{I_M} \\ \frac{-T_x}{I_M} & \frac{M_{mix}}{I_M} & \frac{I_M^z - I_M^y}{I_M} \end{pmatrix}$$

In terms of the magnetic interaction vector components (we restore here the ⊥ symbol) $\mathbf{M}_\perp = (0, M_{\perp y}, M_{\perp z})$

$$P_{mag} = \begin{pmatrix} -1 & 0 & 0 \\ \frac{2\,\mathrm{Im}(M_{\perp y} M_{\perp z}^*)}{\mathbf{M}_\perp \mathbf{M}_\perp^*} & \frac{M_{\perp y}^2 - M_{\perp z}^2}{\mathbf{M}_\perp \mathbf{M}_\perp^*} & \frac{2\,\mathrm{Re}(M_{\perp y} M_{\perp z}^*)}{\mathbf{M}_\perp \mathbf{M}_\perp^*} \\ \frac{2\,\mathrm{Im}(M_{\perp y} M_{\perp z}^*)}{\mathbf{M}_\perp \mathbf{M}_\perp^*} & \frac{2\,\mathrm{Re}(M_{\perp y} M_{\perp z}^*)}{\mathbf{M}_\perp \mathbf{M}_\perp^*} & \frac{M_{\perp z}^2 - M_{\perp y}^2}{\mathbf{M}_\perp \mathbf{M}_\perp^*} \end{pmatrix}$$

Or in terms of the real an imaginary components of the magnetic interaction vector

$$\mathbf{M}_\perp = \mathbf{A} + i\mathbf{B} = (0, A_y, A_z) + i(0, B_y, B_z)$$

$$
P_{mag} = \begin{pmatrix} -1 & 0 & 0 \\ \dfrac{2(A_y B_z - A_z B_y)}{\mathbf{M}_\perp \mathbf{M}_\perp^*} & \dfrac{A_y^2 + B_y^2 - A_z^2 - B_z^2}{\mathbf{M}_\perp \mathbf{M}_\perp^*} & \dfrac{2(A_y A_z + B_y B_z)}{\mathbf{M}_\perp \mathbf{M}_\perp^*} \\ \dfrac{2(A_y B_z - A_z B_y)}{\mathbf{M}_\perp \mathbf{M}_\perp^*} & \dfrac{2(A_y A_z + B_y B_z)}{\mathbf{M}_\perp \mathbf{M}_\perp^*} & -\dfrac{A_y^2 + B_y^2 - A_z^2 - B_z^2}{\mathbf{M}_\perp \mathbf{M}_\perp^*} \end{pmatrix}
$$
